# Next-generation sequencing yields a nearly complete mitochondrial genome of *Plestiodon liui* (Reptilia, Squamata, Scincidae) endemic to China

**DOI:** 10.1080/23802359.2020.1832594

**Published:** 2020-11-03

**Authors:** Na Wu, Bo Cai, Minli Chen, Xianguang Guo

**Affiliations:** a Chengdu Institute of Biology, Chinese Academy of Sciences, Chengdu, China; b University of Chinese Academy of Sciences, Beijing, China; c Key Laboratory of Southwest China Wildlife Resources Conservation (Ministry of Education), China West Normal University, Nanchong, China

**Keywords:** Mitogenome, high-throughput sequencing, phylogenetic tree, skink

## Abstract

The Liu’s skink, *Plestiodon liui*, is endemic to China. In this study, a nearly complete mitochondrial genome (mitogenome) (17,643 bp in length) of *P. liui* from Junzhang Mountain, Wuxi, Jiangsu province, China, was determined by next-generation sequencing. Similar to the typical mtDNA of vertebrates, it contained two ribosomal RNA genes, 13 protein-coding genes (PCGs), 22 transfer RNA genes, and one control region (CR). With exception to the CR, all of the 37 genes were completely recovered. The PCGs were used to perform Bayesian phylogenetic analyses together with other scincid lizards with mitogenome data in GenBank. The resulting phylogenetic tree supported the monophyly of the *P. capito* group, and suggested that *P. liui* is the sister taxon to *P. capito* plus *P. tunganus*. The mitogenome of *P. liui* will provide fundamental data for the exploration of the mitogenome evolution in skinks.

In this study, we determined the mitochondrial genome (mitogenome) of the Liu’s skink, *Plestiodon liui*, a blue-tailed skink endemic to China (Hikida and Zhao 1989), by using the next-generation of sequencing, with GenBank accession number MT662111. The specimen (voucher number CB202004001) was collected from Junzhang Mountain (31.442742˚N, 120.223861˚E), Wuxi city, Jiangsu province, China on 1 April 2020. Its liver tissue was fixed with 95% ethanol, and stored at −20 °C in the herpetological collection, Chengdu Institute of Biology, Chinese Academy of Sciences. A small amount of liver tissue was shipped to Sangon Bitotech (Shanghai, China) for genomic extraction and 150-base-pair paired-end library construction; sequencing was performed on an Illumina Hiseq 2000 instrument (Illumina, San Diego, CA). De novo assembly of clean reads was performed using SPAdes v3.11.0 (Bankevich et al. 2012). The mitogenome of *Pleistiodon tunganus* (MK370739; Chen et al. 2019) was further used as a reference to assemble that of *P. liui*. The sequence was annotated with the MITOS (Bernt et al. 2013) webserver; meanwhile, the complete mitogenomes of some other skink species available in GenBank were also used as reference points for annotations.

The nearly complete mitogenome of *P. liui* is 17,643 bp in length, which is composed of 24.7% (T), 29.6% (C), 30.5% (A), 15.2% (G). The mitogenome of *P. liui* is similar to the typical vertebrate mitochondrial genome. It encodes 37 genes, containing 22 transfer RNA genes (tRNAs), two ribosomal RNA genes (*12S rRNA* and *16S rRNA*), 13 protein-coding genes (PCGs), and a control region (CR or D-loop). In the nearly complete mitogenome of *P. liui*, most of the genes are distributed on the H- strand, except for the *ND6* subunit gene and 8 tRNA genes (*tRNA-Gln, Ala, Asn, Cys, Tyr, Ser^[UCN]^, Glu,* and *Pro*), which are distributed on the L-strand. In the 13 PCGs, 12 PCGs are initiated with the typical ATG codon, only *COX1* with GTG; three PCGs (*ND1, ND2, Cytb*) use TAG as stop codon; four PCGs (*ATP8, ATP6, ND4L, ND5*) use TAA as stop codon; *COX1* and *ND6* use AGA and AGG as stop codons, respectively; the others (*COX2, COX3, ND3, ND4*) terminate with T as an incomplete stop codon. As for the CR, 375 bp were already determined adjacent to *tRNA-Pro*, along with 1851 bp prior to *tRNA-Phe*.

The concatenated PCGs of Scincidae available in GenBank and *Smaug warren* (Cordylidae) as outgroup were used to reconstruct the Bayesian phylogenetic tree for assessing mitochondrial sequence authenticity of *P. liui* and its phylogenetic placement. DNA sequences were aligned in MAFFT v7.313 (Katoh and Standley 2013). The PCGs were translated into amino acid sequences, and then all sequences were concatenated into a nucleotide data set (11,388 bp in total) through PhyloSuite v1.2.1 (Zhang et al. 2020). The plug-in programs in PhyloSuite were used for gene partitioning, model selection, and tree reconstruction. The best-fitting substitution models and partitioning schemes were selected in PartitionFinder v2.1.1 using the Bayesian information criterion (Lanfear et al. 2017). Partitioned Bayesian analyses were conducted using MrBayes v.3.2.6 (Ronquist et al. 2012). As shown in Figure 1, the phylogenetic tree recovered the monophyly of Scincidae and the *P. capito* species group (Brandley et al. 2011, 2012), and suggested that *P. liui* is the sister taxon to *P. capito* plus *P. tunganus* (Cai et al. 2019). The mitogenome of *P. liui* will provide fundamental data for the exploration of the mitogenome evolution in skinks (Scincidae).

**Figure 1.**
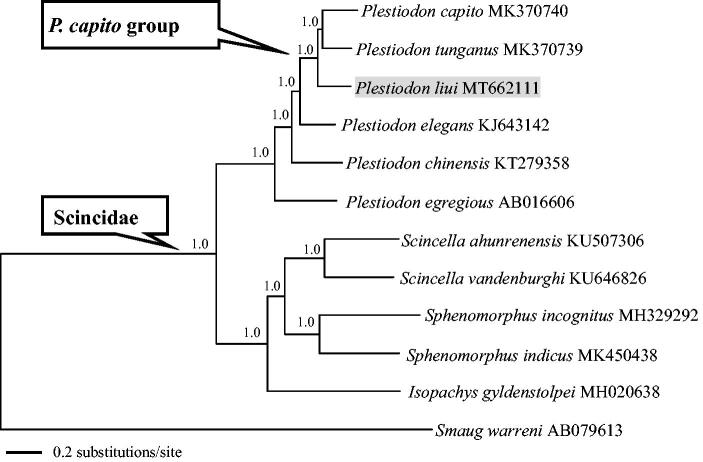
A majority-rule consensus tree inferred from Bayesian inference using MrBayes with the best models for each partition, based on the PCGs of 11 species of Scincidae and one outgroup. The phylogenetic placement of *P. liui* is highlighted. GenBank accession numbers are given with species names. Node numbers show Bayesian posterior probabilities. Branch lengths represent means of the posterior distribution.

## Data Availability

The data that support the findings of this study are openly available in NCBI at https://www.ncbi.nlm.nih.gov/, reference number [MT662111], or available from the corresponding author.

## References

[CIT0001] Bankevich A, Nurk S, Antipov D, Gurevich AA, Dvorkin M, Kulikov AS, Lesin V, Nikolenko S, Prjibelski A, Pyshkin A, et al. 2012. SPAdes: a new genome assembly algorithm and its applications to single-cell sequencing. J Comput Biol. 19(5):455–477. 22506599 10.1089/cmb.2012.0021 PMC3342519

[CIT0002] Bernt M, Donath A, Jühling F, Externbrink F, Florentz C, Fritzsch G, Pütz J, Middendorf M, Stadler PF. 2013. MITOS: improved de novo metazoan mitochondrial genome annotation. Mol Phylogenet Evol. 69(2):313–319. 22982435 10.1016/j.ympev.2012.08.023

[CIT0003] Brandley MC, Ota H, Hikida T, de Oca ANM, Ferıa-Ortız M, Guo X, Wang Y. 2012. The phylogenetic systematics of blue-tailed skinks (*Plestiodon*) and the family Scincidae. Zool J Linn Soc. 165(1):163–189.

[CIT0004] Brandley MC, Wang Y, Guo X, de Oca ANM, Ferıa-Ortız M, Hikida T, Ota H. 2011. Accommodating heterogenous rates of evolution in molecular divergence dating methods: an example using intercontinental dispersal of *Plestiodo*n *(Eumeces*) lizards. Syst Biol. 60(1):3–15. 20952756 10.1093/sysbio/syq045

[CIT0005] Cai B, Yao M, Guo X. 2019. First record of *Plestiodon liui* from Henan province, China and analysis of its phylogenetic placement. Chin J Zool. 54:668–677.

[CIT0006] Chen M, Liu J, Chen D, Guo X. 2019. The complete mitochondrial genome of a blue-tailed skink (*Plestiodon tunganus*) endemic to Sichuan Basin. Mitochondrial DNA B. 4(1):1109–1110.

[CIT0007] Hikida T, Zhao E. 1989. *Eumeces liui*: a new species of blue-tailed skink (Lacertilia: Scincidae) from China. Copeia. 1989(1):110–114.

[CIT0008] Katoh K, Standley DM. 2013. MAFFT multiple sequence alignment software version 7: improvements in performance and usability. Mol Biol Evol. 30(4):772–780. 23329690 10.1093/molbev/mst010 PMC3603318

[CIT0009] Lanfear R, Frandsen PB, Wright AM, Senfeld T, Calcott B. 2017. PartitionFinder 2: new methods for selecting partitioned models of evolution for molecular and morphological phylogenetic analyses. Mol Biol Evol. 34(3):772–773. 28013191 10.1093/molbev/msw260

[CIT0010] Ronquist F, Teslenko M, Mark PVD, Ayres DL, Darling A, Höhna S, Larget B, Liu L, Suchard MA, Huelsenbeck JP. 2012. MrBayes 3.2: efficient Bayesian phylogenetic inference and model choice across a large model space. Syst Biol. 61(3):539–542. 22357727 10.1093/sysbio/sys029 PMC3329765

[CIT0011] Zhang D, Gao F, Li WX, Jakovlić I, Zou H, Zhang J, Wang GT. 2020. PhyloSuite: an integrated and scalable desktop platform for streamlined molecular sequence data management and evolutionary phylogenetics studies. Mol Ecol Resour. 20(1):348–355. 31599058 10.1111/1755-0998.13096

